# Isolation, Characterization, and Genomic Analysis of Bacteriophages Against *Pseudomonas aeruginosa* Clinical Isolates from Early and Chronic Cystic Fibrosis Patients for Potential Phage Therapy

**DOI:** 10.3390/microorganisms13030511

**Published:** 2025-02-26

**Authors:** Hanzada T. Nour El-Din, Maryam Kettal, José C. Granados Maciel, Greg Beaudoin, Umut Oktay, Sabahudin Hrapovic, Subash Sad, Jonathan J. Dennis, Danielle L. Peters, Wangxue Chen

**Affiliations:** 1 Department of Immunobiology, Human Health Therapeutics Research Center, National Research Council Canada, Ottawa, ON K1N 5A2, Canada; mkett072@uottawa.ca (M.K.); jose1.granadosm@gmail.com (J.C.G.M.); greg.beaudoin@nrc-cnrc.gc.ca (G.B.); danielle.peters@nrc-cnrc.gc.ca (D.L.P.); wangxue.chen@nrc-cnrc.gc.ca (W.C.); 2 Faculty of Science, Biological Sciences, University of Alberta, Edmonton, AB T6G 2R3, Canada; uoktay@ualberta.ca (U.O.); jon.dennis@ualberta.ca (J.J.D.); 3 Aquatic and Crop Resource Development (ACRD) Research Center, National Research Council Canada, Montreal, QC H4P 2R2, Canada; sabahudin.hrapovic@cnrc-nrc.gc.ca; 4 Department of Biochemistry, Microbiology and Immunology, Faculty of Medicine, University of Ottawa, Ottawa, ON K1N 6N5, Canada; subash.sad@uottawa.ca; 5 Department of Biology, Brock University, St. Catharines, ON L2S 3A1, Canada

**Keywords:** *Pseudomonas aeruginosa*, cystic fibrosis, bacteriophage, lytic phage, antimicrobial resistance

## Abstract

*Pseudomonas aeruginosa* is associated with both community and hospital-acquired infections. It colonizes the lungs of cystic fibrosis (CF) patients, establishing an ecological niche where it adapts and evolves from early to chronic stages, resulting in deteriorating lung function and frequent exacerbations. With antibiotics resistance on the rise, there is a pressing need for alternative personalized treatments (such as bacteriophage therapy) to combat *P. aeruginosa* infections. In this study, we aimed to isolate and characterize phages targeting both early and chronic *P. aeruginosa* isolates and evaluate their potential for phage therapy. Four highly virulent phages belonging to myoviral, podviral, and siphoviral morphotypes were isolated from sewage samples. These phages have a broad host range and effectively target 62.5% of the *P. aeruginosa* isolates with a positive correlation to the early isolates. All the phages have a virulence index of ≥0.90 (0.90–0.98), and one has a large burst size of 331 PFU/cell and a latency period of 30 min. All phages are stable under a wide range of temperature and pH conditions. Genomic analysis suggests the four phages are strictly lytic and devoid of identifiable temperate phage repressors and genes associated with antibiotic resistance and virulence. More significantly, two of the phages significantly delayed the onset of larval death when evaluated in a lethal *Galleria mellonella* infection model, suggesting their promise as phage therapy candidates for *P. aeruginosa* infections.

## 1. Introduction

Antimicrobial resistance (AMR) is a critical global health issue, rendering previously effective antibiotics powerless against infectious organisms. In 2019, AMR accounted for 1.2 million deaths, and it currently causes 700,000 deaths annually, with predictions reaching up to 8.2 million deaths by 2050 [1,2]. When bacteria are exposed to antibiotics, a natural phenomenon of resistance occurs due to the selection pressure exerted on susceptible bacteria, killing or inhibiting them, while bacteria that are intrinsically resistant to the drugs or that have acquired resistance are more likely to survive. Antibiotic resistance is exacerbated not just by the misuse of antibiotics but also by their inappropriate use (insufficient dosage and non-compliance with treatment protocols). Currently, our capacity to treat infectious diseases is severely compromised by the surge in resistance, which has detrimental effects on health outcomes like prolonged illness, higher medical expenses, and increased mortality rates [3].

Among the multidrug-resistant (MDR) pathogens, six are known as the ESKAPE pathogens (*Enterococcus faecium*, *Staphylococcus aureus*, *Klebsiella pneumoniae*, *Acinetobacter baumannii*, *Pseudomonas aeruginosa*, and *Enterobacter* spp.), which are commonly spread in hospitals and are becoming highly resistant to last-line antibiotics [4]. *P. aeruginosa* is particularly concerning, as it accounts for 16.2% of all intensive care unit (ICU) infections and 23% of ICU-acquired infections worldwide, with mortality rates reaching up to 30% within 30 days in patients who experienced delayed appropriate treatment for more than 52 h [5,6,7]. A major group affected by *P. aeruginosa* infections are individuals with cystic fibrosis (CF). These infections are commonly polymicrobial, yet approximately 49.6% of CF patients are specifically infected with *P. aeruginosa*, increasing to 74.1% among those over 26 years of age [8,9]. This organism’s biofilm formation ability, which shields it from antibiotics and the host immune response, combined with its adaptability to oxidative stress in CF lungs, lead to persistent infections that are difficult to eradicate [10]. Moreover, the stressful CF conditions encountered by *P. aeruginosa* lead to mutations, and those mutant isolates are selected during the adaptation phase that marks the shift from early (acute) to chronic stages [11,12,13]. For example, changes are evident in the *algU mucABCD* gene cluster encoding the *algU* gene responsible for alginate production and in the *mucA*, *mucB*, or *mucD* genes that act as negative regulators of AlgU [14]. Alginate is a thick mucopolysaccharide slime layer that contributes to biofilm formation, protects against host immune responses, and increases antibiotic resistance. In chronic serious CF infections, constitutive mucoid phenotype isolates are predominant. Those isolates have mutations in the alginate negative regulators, leading to increased alginate production [15,16]. As the infection progresses, an inflammatory response is triggered that leads to lung tissue destruction.

Despite advances in antibiotic treatments, the persistence and MDR capabilities of *P. aeruginosa* strains necessitate novel therapeutic strategies. Bacteriophages, or phages, are viruses that specifically infect bacteria [17]. They are ubiquitous in nature and found in environments like oceans, soil, and the human body [18]. An amenable feature of phages is their high specificity, a characteristic that prevents dysbacteriosis and avoids side effects typically associated with broad-spectrum antibiotics [19]. Additionally, phages show great potential in treating biofilms. This is attributed to their small size and phage-encoded depolymerases, which are enzymes that aid in the degradation of a main biofilm component called extracellular polysaccharide [20].

The escalating threat of AMR, particularly in *P. aeruginosa* strains affecting CF patients, underscores the need for innovative treatments like phage therapy. This approach offers a targeted, effective alternative to traditional antibiotics, potentially transforming the management of MDR bacterial infections for CF patients.

The purpose of this study is to isolate and characterize phages against *P. aeruginosa* isolates from CF patients and test the phage–bacterial host dynamics.

To that end, we isolated four lytic *P. aeruginosa* phages—U17 “vB_PaeP_HTN1”, AA17 “vB_PaeS_HTN2”, AA20 “vB_PaeM_HTN3”, and AC20 “vB_PaeM_HTN4”—that show high virulence against CF *P. aeruginosa* isolates. We characterized their phenotypic, morphological, and genomic characteristics. We also tested the difference in the interplay between the phages and early or chronic *P. aeruginosa* isolates from CF patients. Knowledge about such dynamics plays a pivotal role in the success of phages when used as therapeutic agents for such infections.

## 2. Materials and Methods

### 2.1. Bacterial Strains and Culture Conditions

Fifty-six *P. aeruginosa* strains collected from CF patients [21] were used (Table S1). The strains were categorized as either early or chronic. Early isolates (n = 30) were obtained from the patients’ first positive sputum samples, while chronic isolates (n = 26) were collected from patients who had a history of positive sputum cultures spanning four years. Strains were grown aerobically overnight at 37 °C on Lennox Luria Bertani (NaCl, 5 g/L tryptone, 10 g/L, yeast extract, 5 g/L) “LLB” agar plates or in LLB broth with shaking at 200 rpm. For phage assays, LLB broth was supplemented with 10 mM CaCl_2_ and MgCl_2_ “LLB-S”.

### 2.2. Midi-Throughput Phage Hunting

#### 2.2.1. Sewage Sample Preparation

Sewage samples received from the University of Ottawa’s Environmental Engineering laboratory were passed through a cell strainer of 40–70 µm to remove any debris, centrifuged, and filter-sterilized using a 0.22 µm polyethersulfone (PES) membrane filter (MilliporeSigma, Burlington, MA, USA). Samples were then concentrated ~5× using a 100 kDa MWCO centrifugal filter (MilliporeSigma, Burlington, MA, USA) and stored at 4 °C.

#### 2.2.2. Phage-Hunting Plate Assay

**Figure 1:**
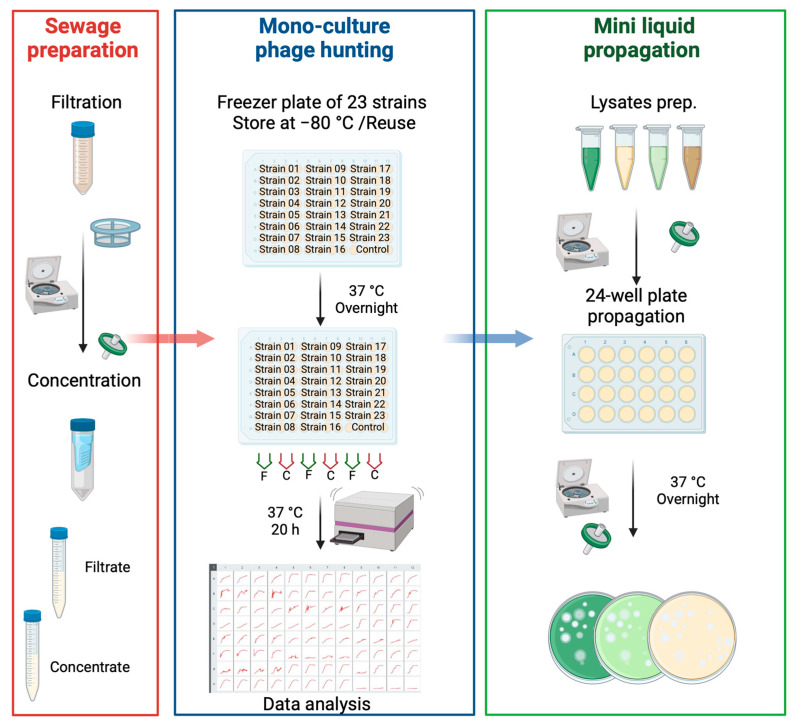
Midi-throughput phage hunting. Overview of the workflow used for phage hunting. Stage 1: sewage sample preparation through filtration and concentration. Stage 2: stocking of bacterial isolates in 96-well plates, preparation of hunting plates with both bacterial isolates and sewage samples, incubation, and data analysis for the presence of potential phages. Stage 3: mini liquid propagation, followed by testing for potential phage isolation by the double agar layer assay. Abbreviations: F = filtrate, C = concentrate. The figure was created with BioRender.com.

A set of 23 *P. aeruginosa* isolates (13 early and 10 chronic isolates) was used (Figure 1). Each isolate was propagated overnight in LLB, followed by the addition of 20% glycerol. Quadruplicate wells of each isolate were prepared in a 96-well plate and stored at −80 °C. The freezer plate was then used for multiple rounds of phage hunting. A multichannel pipette with sterile tips was used to scrape and transfer a small number of frozen cells into 200 µL LLB in the corresponding wells of a fresh 96-well plate. This plate was incubated overnight. Then, 95 µL each of sewage sample concentrate and 2X LLB-S were aliquoted into a 96-well plate, to which 10 µL of overnight bacterial culture was added. Sterile sewage filtrate was used as a negative control. The plates were then incubated in a LogPhase 600 spectrophotometer (Agilent, Santa Clara, CA, USA) at 37 °C and 800 rpm shaking, with OD_600_ measurements taken every 20 min for 20 h. The following day, growth curve data were analyzed, and wells that may contain potential phages were identified by comparing the growth curves of the untreated control wells to the sewage-spiked groups.

Lysates with potential phages were centrifuged, filter-sterilized, and subjected to a mini-liquid propagation step to amplify any potential phages. Briefly, 100 µL of potential phage lysate was pre-incubated with 100 µL of the host sub-culture for 10 min at room temperature (RT) in a 24-well tissue culture plate. Then, for each potential phage/host co-culture, 2 mL LLB-S was added and incubated overnight. On the following day, the co-cultures were centrifuged, filter-sterilized using a 0.22 µm PES membrane filter, and tested for the presence of phages against the corresponding host by the double agar layer overlay assay (Figure 1).

The potential isolated phages were subjected to three rounds of purification, and stocks of the isolated phages were then prepared by either liquid or solid-plate propagation techniques on the appropriate bacterial host [22].

### 2.3. Phage Phenotypic and Genomic Characterization

#### 2.3.1. Host Range, Phage–Host Interaction Data Analyzer (PHIDA) and Efficiency of Plating (EOP)

Host range determination was performed on all the isolated phages against the full panel of clinical isolates using the spot testing assay [23]. The purified phage lysates were standardized to ~10^9^ plaque-forming units/mL (PFU/mL) and spotted in triplicates on LLB plates inoculated with overnight culture from each tested strain. Plates were then incubated at 37 °C for 16 h, and on the following day, plaques were scored visually based on their clarity, as follows: +4 is complete clearing, +3 is clearing with a faintly hazy background, +2 is substantial turbidity through the cleared zone, +1 is few individual plaques, and 0 is no clearing [23,24].

Host ranges of the four leading phages that covered most of the isolates’ panel were also tested using a liquid-based turbidimetric assay [25]. The plate layout was set up as described by Martinez-Soto et al. using a 384-well plate. A single plate can test up to four phages in triplicate against 22 bacterial hosts, including a growth control (host + Trypticase soya broth “TSB”) and sterility controls (phage + TSB and SM buffer “100 mM NaCl, 25 mM Tris-HCl pH 7.5, 8 mM MgSO4” + TSB). Equal volumes of both the tested strains and standardized phage lysates were used to reach a final theoretical multiplicity of infection (MOI) of 1. Plates were then incubated at 37 °C for 24 h with orbital continuous shaking at 180 rpm using the BioTek Synergy H1 microplate reader (Agilent, Santa Clara, CA, USA), and readings at OD_600_ were measured every 30 min. Data were then obtained from the plate reader and analyzed using the PHIDA analyzer sheet [25].

The EOP was carried out on the same four phages, as previously described [26], by preparing an overlay of the isolate of interest and spotting tenfold serial dilutions of the tested phage on the lawn. Combinations were determined based on the susceptible strains from the host range spot assay testing, where two biological replicates and three technical triplicates were produced for each *P. aeruginosa* isolate and phage pair. EOP was calculated as the titer that a phage produces on a bacterial strain compared to the maximum titer observed on its isolation host strain. The EOP was classified into four levels of efficiency, from “high production” to “no production”, as follows: high (≥0.5–1), medium (<0.1–0.5), low (≤0.001–0.1), and no production (≤0.001) [27]. Additionally, when a phage was able to infect and lyse the host but could not produce individual plaques or plaques were faint to count at the appropriate dilution, it was given a designation of II (Table S2).

#### 2.3.2. CsCl Gradient Purification and Transmission Electron Microscopy (TEM)

The four tested phages were purified for TEM imaging using CsCl density gradient centrifugation. For every phage, phage lysate—at least 10^10^ PFU/mL—was precipitated using the zinc chloride method, as previously described [28]. The precipitate was resuspended in 16 mL of SM buffer, solid CsCl was added at a rate of 0.75 g/mL of phage suspension, and the rest of the protocol was performed according to the manufacturer recommendations (Beckman Coulter, Brea, CA, USA, LE-80 Ultracentrifuge). The CsCl gradient was centrifuged at 40,000 rpm in a 70.1 Ti rotor at 4 °C for 20 h. For each phage, the ghost band was removed and washed with SM buffer [29].

Transmission electron microscopy analysis (TEM HITACHI H-7500, Tokyo, Japan), equipped with a bottom-mounted AMT NanoSprint 12 MP camera and operating at 80 kV in high-contrast mode, was performed using a negative-staining technique. TEM grids (Cu 200 mesh, 15–25 nm carbon-supported, Ted Pella Inc., Redding, CA, USA) were freshly glow-discharged using EMS GloQube-D, a dual-chamber glow discharge system (Electron Microscopy Sciences, Hatfield, PA, USA), in negative mode with a plasma current of 25 mA for 45 s. Such grids were floated on a 15 µL sample aliquot for 60 s. The excess liquid was wicked away from the edge of the grid with a filter paper strip (Whatman 541, Maidstone, UK). The grid was then rinsed three times with droplets of double distilled water and stained in 1% uranyl acetate for 60 s. The stain was removed using a fresh piece of filter paper, and the grid was dried at ambient conditions for 2 h. The dimensions of the virions were measured by ImageJ version 1.53 (National Institutes of Health, Bethesda, MD, USA) with reference to the scale bar generated from the microscope.

#### 2.3.3. Phage Stability Assays

The pH stability of the four leading phages was tested over a broad pH spectrum, ranging from 3 to 11. The SM buffer pH values were adjusted by using either 0.2 N hydrogen chloride (HCl) or 0.5 M sodium hydroxide (NaOH). The phage lysates were standardized at ~10^9^ PFU/mL, except for AC20 (~10^8^ PFU/mL), then diluted 1:100 in low-bind PCR tubes using the adjusted SM buffers and incubated for 1 h at 4 °C. At the end of the experiment, the phage titers were measured by serially diluting the phage lysates tenfold and spotting all dilutions on a bacterial lawn of the phage’s propagation host. Plates were incubated at 37 °C overnight, and on the following day, titers were counted, and PFUs were calculated.

To test the thermal stability, aliquots of the four standardized phage stocks (10^9^ PFU/mL) were dispensed into low-bind PCR tubes and incubated for 1 h at 50 °C, 60 °C, 70 °C, 80 °C, and 90 °C. At the end of the experiment, samples were allowed to cool down to RT, and phage titers were measured as described earlier. The thermal inactivation point is defined as the temperature at which the phage completely loses its lytic activity.

For shelf-life stability testing, phage lysates were standardized to ~10^10^ PFU/mL, except for U17 (10^9^ PFU/mL), and kept at 4 °C. Titers were checked monthly for the first three months and then on the sixth month. All stability assays were performed on three biological replicates and spotted in three technical replicates.

#### 2.3.4. Phage Genomic Analysis and Restriction Fragment Length Polymorphism

For the four phages, the DNA extraction was performed using the phenol–chloroform–isoamyl alcohol method, as previously detailed, with the modification of using the ZnCl_2_ precipitation method at the start of the procedure [22]. Briefly, 1 mL of reaction buffer (10 mM CaCl_2_ and 250 mM MgCl_2_), 10 µL of DNase (2000 U/mL), and 10 µL of RNase (10 mg/mL) were added to 10 mL of phage lysate (10^10^ PFU/mL) and left for 60 min at 37 °C. Then, we added 40 mM ZnCl_2_ and incubated at 37 °C for 5 min with shaking. The mixture was then centrifuged at 8000× *g* for 10 min, and the pellet was resuspended overnight in 1 mL of SM buffer. For DNA extraction, the supernatant for each phage from the previous step was processed as per the reference protocol. At the end of the experiment, the purity and concentration of obtained DNA were checked with a NanoDrop ND-1000 spectrophotometer (Thermo Scientific, Waltham, MA, USA) and Qubit Fluorometer (Thermo Scientific, Waltham, MA, USA) and run on a 0.7% TAE gel to check the gDNA integrity.

Restriction fragment length polymorphism (RFLP) analysis was performed as previously described [22] on 1 µg of each of the four tested phage DNA and *Escherichia* phage Lambda gDNA as the experimental control. A panel of 16 restriction enzymes—EcoRI, BamHI, BgIII, Eco321, HindIII, KpnI, SmaI, PstI, SaII, NdeI, NotI, Xhol, XbaI, MspI, HpaI, and PaeI (FastDigest, Thermo Scientific, Waltham, MA, USA)—were added, as per the manual’s instructions, to the DNA. Reactions and uncut controls were separated on a 1.2% agarose gel and imaged on a ChemiDoc system (Bio-Rad, Hercules, CA, USA).

The DNA was standardized and sent to Genome Quebec for library creation using Covaris and sequencing on an Illumina NovaSeq (Illumina, Inc., San Diego, CA, USA) for paired-end sequencing. The paired reads were trimmed with BBDuk Trimmer v.1.0 for all adapter sequences and minimum Q20 ends, with short reads (<10 bp) discarded. The reads were merged with BBMerge v38.84, and duplicate reads were removed with Dedupe v38.84 using kmer seed length 31, with no substitutions or edits. The processed reads underwent de novo SPAdes v.3.15.5 [30] assembly using the following parameters: multicell, error correct and assemble, and careful mode with 5% of the reads for both AA17 and U17 and 1% of the reads for AA20 and AC20. Reads were re-aligned to each contig using Bowtie2 v.2.4.5 [31] with the following parameters: end-to-end and medium sensitivity, with only the best match reported.

The open reading frames (ORFs) were called using the Glimmer3 plugin (v1.5) [32] for Geneious (2025.0.2), PHANOTATE [33], and Geneious ORF finder, then manually curated. Putative tRNAs were identified using tRNAscan-SE v.2.0.5 [34,35]. Protein functions were assigned based on InterProScan-SE [36] results from the following applications: CDD, Coils, Gene3d, HAMAP, MobiDB-Lite, Panther, PfamA, Phobius, PIRSF, PRINTS, PrositePatterns, PrositeProfiles, SFLD, SignalP, SignalP_EUK, SignalP_GRAM_NEGATIVE, SMART, SuperFamily, TIGRFAM, and TMHMM. Geneious Annotate by BLASTx was used to further annotate the genome using the nucleotide collection (nr/nt) with five hits maximum and a similarity cut-off of 75%. The BLOSUM62 matrix was used with 11 1 gap cost, word size of 6, and max E-value of 0.05. Finally, the annotated genomes were compared to their closest relatives in the NCBI database to set the genome origin and assess taxonomy. Further analysis of phages U17 and AA17 was completed using the conserved large terminase protein from each phage against the top 100 BLASTp hits from the NCBI database. A Clustal Omega (v 1.2.3) [37] protein alignment with fast clustering and 100 mBed guide trees was performed with the BLASTp hits, and the resulting alignment was run with RAxML (v8.2.11) to generate a phylogenetic tree [38]. The RAxML settings include the GAMMA BLOSUM62 protein model, with rapid hill-climbing with RELL and 1000 replicates.

Genomes were submitted to NCBI with the following accession numbers: *Pseudomonas* phage vB_PaeP_HTN1 (U17); PP916318, *Pseudomonas* phage vB_PaeS_HTN2 (AA17); PP916319, *Pseudomonas* phage vB_PaeM_HTN3 (AA20); PP916316, and *Pseudomonas* phage vB_PaeM_HTN4 (AC20); PP916317.

### 2.4. Single Burst Size Analysis “One-Step Growth Curve”

The one-step growth curves for the four phages were performed as previously described [39], with minor modifications. Time points and full experiment length were based on pilot experiments. Briefly, for every phage, the corresponding bacterial host’s overnight culture was diluted to an OD_600_ of 0.2 and grown to a mid-exponential phase (OD_600_ = 0.5), corresponding to ~1 × 10^8^ CFU/mL. At this point, an appropriate volume of the tested phage was added to achieve an MOI of 0.001. This was considered the adsorption tube, which was incubated at RT for 5 min to allow phage to attach. Following the adsorption step, the phage–bacteria mixture was diluted 1:100 and further incubated at 37 °C with shaking at 180 rpm. Aliquots were removed at different time points, and phage titers were determined by either serial dilution and spot-plating using the double agar layer plates or by the full plate overlay assay. Burst size was calculated using the formula “burst size = P/I”, where P is the maximum number of phages after lysis, and I is the initial phage number added to the culture.

### 2.5. In Vitro and In Vivo Virulence Assays

#### 2.5.1. Growth Inhibition Curves

The kill curves for the four tested phages against the corresponding bacterial hosts were performed as previously described [29,40,41] with minor modifications. Briefly, 100 µL aliquots of bacterial subcultures, corresponding to ~10^7^ CFU/mL, were mixed with 100 µL of different dilutions of the tested phages to achieve variable MOIs in a 96-well plate. The plate was incubated in a LogPhase 600 spectrophotometer (Agilent, Santa Clara, CA, USA) at 37 °C with 800 rpm orbital shaking, and the OD_600_ was measured hourly for 20 h. Experiments were performed on three biological replicates and spotted in three technical replicates. The virulence index was calculated during exponential growth, with the data cut-off at the start of the stationary phase, which lies between 13 and 16 h, depending on the control bacterial host of the respective phage tested. Local virulence (VL) was calculated by dividing the area under the curve (AUC) for each of the tested MOIs by the AUC of the control bacterial host. Then, the obtained value was subtracted from 1 to determine the VL for each MOI. The global virulence index (Vi) was calculated by averaging the AUC obtained from plotting the AUC of each MOI VL against log10 MOI.

#### 2.5.2. *Galleria mellonella* Phage Rescue Experiments

*Galleria mellonella* infections were conducted as previously described, with several changes [42]. Overnight cultures were set up in LLB using a population of 3–4 bacterial colonies. Cells were subcultured 1:50 and grown to an OD_600_ of 0.3–0.5, washed once with SM buffer, and then resuspended in SM. Cells and phages were further diluted in SM to reach the desired CFU or PFU/mL. *G. mellonella* larvae were cultured at 30 °C with a diet composed of the following ratios: 264 g of wheat germ, 132 g of brewer’s yeast, 210 g of beeswax, 132 g of glycerol, 132 g of honey, and 66 g of water. Larvae weighing between 225 and 275 mg were selected for injections. Then, 5 µL of the diluted bacterial suspension was injected into the right proleg of the worm with a 250 µL Hamilton syringe, followed by an injection of the corresponding phage sample into the left proleg an hour after the bacterial administration. SM buffer was selected as the negative control for infections. Larvae were placed in a static 37 °C incubator following the injections, and the survival of the larvae was monitored every 8 h after the 16 h period for a total of 48 h. Larvae were recorded as “dead” if no response to touch was observed. The statistical analysis was performed using the Gehan–Breslow–Wilcoxon test.

### 2.6. Bacterial Twitching Assay

To further investigate possible correlations between phage infection efficiency and stage of *P. aeruginosa* disease (early vs. chronic), a twitching motility assay of the 56 *P. aeruginosa* strains was conducted as previously described [43], with some modifications. The medium was prepared on the same experiment day as follows: 20 g Eiken Agar, 2.8 g tryptone, 1.4 g yeast extract, and 1.4 g NaCl per L. Plates were then poured directly on a well-leveled surface and allowed to dry. Diluted overnight bacterial cultures were grown to an OD_600_ of 2.0. At this point, 10 µL of this culture was gently stabbed vertically into the plate through the bottom for 5 s without expensing the culture. Then, the tip was carefully lifted out of the agar and disposed of. The plates were then incubated at 37 °C overnight, with a tray of water underneath to provide a humid environment. On the following day, the agar was disposed of by plopping it off using a metal spatula, and plates were incubated for 60 min at 50 °C. Finally, the plates were photographed, and twitching motility was quantified by taking measurements of the twitching zone diameters from three independent stabs. The experiment was performed in biological triplicates, with four plates for each replicate.

### 2.7. Phage–Host Correlation Analysis

Potential associations between the isolate type and phage host range using spot test, as well as the PHIDA assay and twitching motility, were tested by Fisher’s exact test. In addition, the potential correlation between these variables (either numeric or those that could be converted into pseudo-numeric values) was tested by Pearson’s correlation and represented as a correlation plot. In all the statistical analyses, *p*-values < 0.05 were considered significant. All analyses were carried out in R-Studio version 1.2.1335.

## 3. Results

### 3.1. Phage Isolation and Host Range Determination

From one round of midi-throughput phage hunting with our sewage samples, six distinct phages were isolated. After three rounds of phage purification, the lysates were propagated, and standard phage lysates were stored for testing. As a preliminary evaluation of the six isolated potential phages, we used the spot test assay, which is the gold standard for evaluating phage infectivity across bacterial strains. The full panel of 56 *P. aeruginosa* isolates were used in this assay. Six phages were able to produce complete clearing zones, indicating infectivity, on 35 isolates, representing 62.5% of the panel (Figure S1A). The early isolates represented 63% of this percentage of infected strains. When the six phages were clustered based on the spot assay results, four of them were clustered separately from the remaining two phages (Figure S1B). Those four phages infected the same percentage of isolates in the spot test when compared to the six phages collectively, and hence, we only moved forward with those phages: U17 “vB_PaeP_HTN1”, AA17 “vB_PaeS_HTN2”, AA20 “vB_PaeM_HTN3”, and AC20 “vB_PaeS_HTN4”. Based on the phage’s ability to infect the tested strains, AA20 was ranked at the top with 33 infected strains, followed by AA17, which infected 14 strains, then AC20 with 12 strains, and finally, U17 with 9 infected strains.

### 3.2. Phenotypic and Genomic Characterization of the Four Potential Phages

#### 3.2.1. EOP and Phage–Host Interaction Data Analyzer (PHIDA)

To test for the plaquing ability of the four phages, EOP was tested on phage-sensitive strains from the spot assay experiment. Strains with an EOP value below 0.001 were considered non-infective to the tested strain. For phages that could infect the host but could not produce individual plaques or when plaques were masked with prophages, no titer was calculated. Phage AA20 demonstrated the highest EOP on many susceptible isolates, while AA17 showed medium and high EOP values, primarily on early isolates (Table S2, Figure S2).

**Figure 2:**
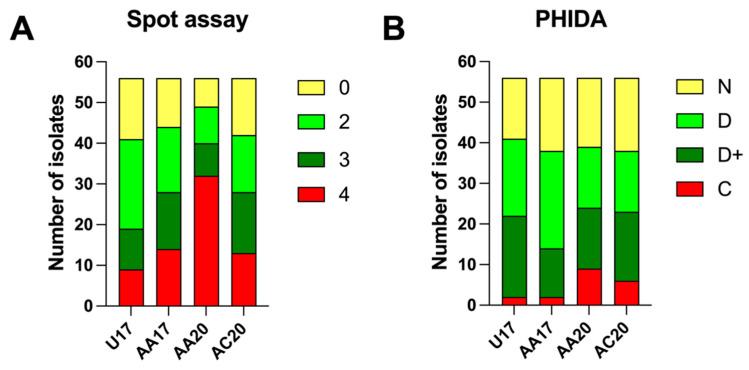
Bar plots showing the spot assay versus PHIDA results. (**A**) Spot assay results. The numbers represent the lysis pattern in the spot: 4 = complete clearing, 3 = clearing with hazy background, 2 = substantial turbidity through the cleared zone, 1 = few individual plaques, and 0 = no clearing. (**B**) Phage–host interaction results expressed as C = complete inhibition of the bacterial growth, D+ = more than 5 h delay in bacterial growth compared to control, D = time difference to reach detection threshold between sample and control is ≥1 and <5 h, and N = small effect on bacterial growth endpoint.

To further examine the phage–host interactions, a PHIDA experiment was conducted in liquid culture media. The results show a pattern similar to that obtained from the spot test. However, the phages’ effect on the susceptible hosts was lower in the PHIDA assay when comparing complete clearing on plates to complete host inhibition in liquid culture (Figure 2).

#### 3.2.2. Transmission Electron Microscopy

**Figure 3:**
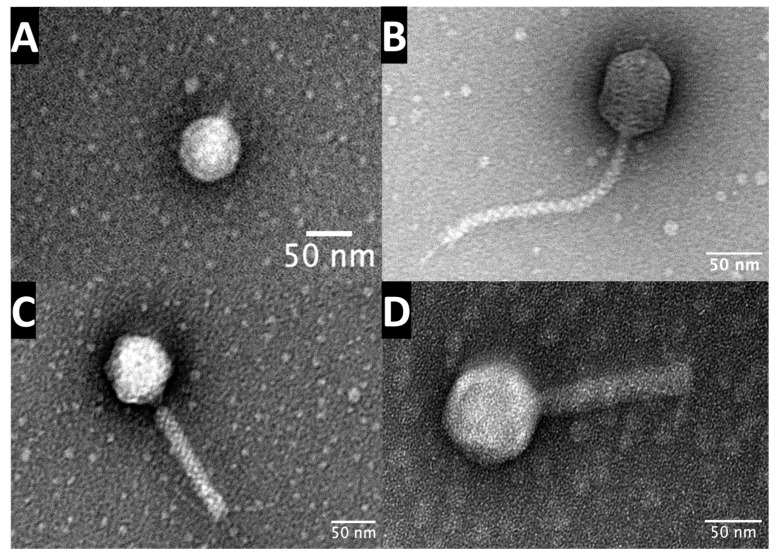
Transmission electron micrographs of the four phages. (**A**) U17 shows a podoviral morphotype, and (**B**) AA17 shows a siphoviral morphotype, while (**C**) AA20 and (**D**) AC20 show a myoviral morphotype. The phages were purified using CsCl density gradient centrifugation, and then for imaging, they were negatively stained with 1% uranyl acetate, and images were captured by TEM HITACHI H-7500 at a scale bar of 50 nm. The virion dimensions were measured by ImageJ v1.53.

The four phages were examined for morphological determination using TEM. Phage U17 is the only one classified as a member of the podoviral morphotype. The head structure is an icosahedron with a length of 61.5 nm ± 3.7 and width of 68 nm ± 3.1. The tail is short and inflexible, with a length of 12 nm (Figure 3). TEM of the AA17 phage shows a siphoviral morphotype featuring an icosahedral capsid with a length and width of 70 nm ± 1.6 and 64 nm ± 3.4, respectively. The tail is long and noncontractile, at 196 nm ± 3.3 long and 10.5 nm ± 0.75 wide. Finally, phages AA20 and AC20 show a myoviral morphotype due to their prolate heads and contractile tails with tail fibers. AA20 capsid’s length and width are 74 ± 4.3 and 65 nm ± 3.2, and its tail length and width are 140 nm ± 1.52 and 16 nm ± 0.49. AC20 capsid’s length and width are 78 ± 2.1 and 69 nm ± 1.8, and its tail length and width are 194 nm ± 2.7 and 10 nm ± 0.3 (Figure 3). All morphological features of the phages are consistent with the assigned morphotypes, according to the ninth report of the International Committee on Taxonomy of Viruses (ICTV) [44].

#### 3.2.3. Stability Studies

A pH Stability

**Figure 4:**
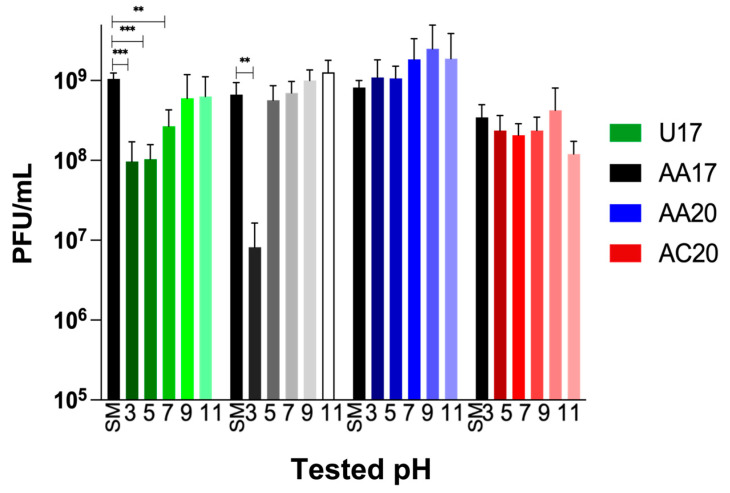
pH stability of the four tested phages. Following an hour of exposure to varying pH levels, phage titers were determined. The pH stability of phages was compared to SM control (pH 7.4). SM control is represented in black, and shades of each color represent pH from low to high. Error bars show the standard deviation of a biological duplicate and technical triplicate experiment. Statistical analysis was performed on each phage set by GraphPad Prism (v9) (GraphPad Software Inc., San Diego, CA, USA) using a one-way ANOVA test, followed by Dunnett’s multiple comparisons test. Statistical significance is represented as ** *p* ≤ 0.01 and *** *p* ≤ 0.001.

Testing phages at various pH levels is crucial for assessing their stability, infectivity, and adaptability in different conditions that the phage might encounter during storage or when used as a therapeutic agent based on the route of administration. Phages AA20 and AC20 show high stability at all the tested pH levels (Figure 4). In contrast, phage U17 is sensitive to pH conditions below 7.5, and the effect is more pronounced with increased acidity, resulting in a log reduction in titer following exposure for 1 h. As for AA17, it shows even higher sensitivity to acidic conditions, with a two-log reduction in titer at a pH of 3. However, it is stable at pH levels of 5 and above (Figure 4).

B Temperature stability

**Figure 5:**
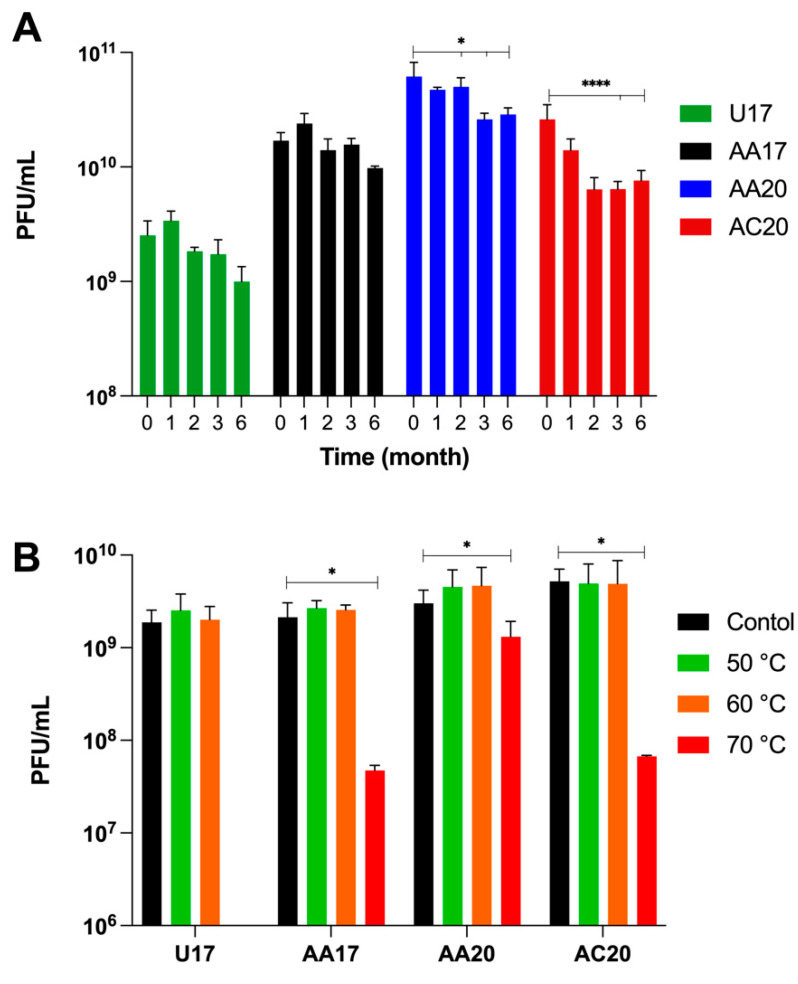
Phage temperature stability. (**A**) The stability of four phages at 4 °C over 6 months. (**B**) The phage stability at variable stressing temperatures (black for control tested at 4 °C). Error bars show the standard deviation of a biological and technical triplicate experiment. Statistical analysis was performed on each phage set by GraphPad Prism (v9) (GraphPad Software Inc., San Diego, CA, USA) using a one-way ANOVA test, followed by Dunnett’s multiple comparisons test. Statistical significance is represented as * *p* ≤ 0.05 and **** *p* ≤ 0.0001.

To assess general stability in normal storage conditions, we monitored the titer of each phage at 4 °C over six months. The four tested phages showed good stability over time, with a maximum of half log reduction in phage AC20 titer (Figure 5A).

We also examined each phage’s stability for one hour at five stressing temperatures from 50 °C to 90 °C (Figure 5B). All phages were stable at 50 °C and 60 °C; however, phage U17 was completely inactivated at temperatures 70 °C and above, while phages AA17 and AC20 had a 1.7 log reduction in titer. Phage AA20 was more stable, with less than half a log reduction at 70 °C (Figure 5B). All phages lost activity following exposure to 80 °C and 90 °C; thus, they are not shown in the figures.

#### 3.2.4. Genomic and RFLP Analysis

A Genomic Analysis and Annotations

The phages were sequenced, the resulting reads were merged and trimmed, and duplicates were removed. The resulting reads were de novo assembled into contigs, and reads were re-aligned to confirm the genome sequences and coverage. The general genome features of the four phages are summarized in Table 1, and the full genome annotations for the four phages are detailed in supplementary Tables S3–S6.

**Figure 6:**
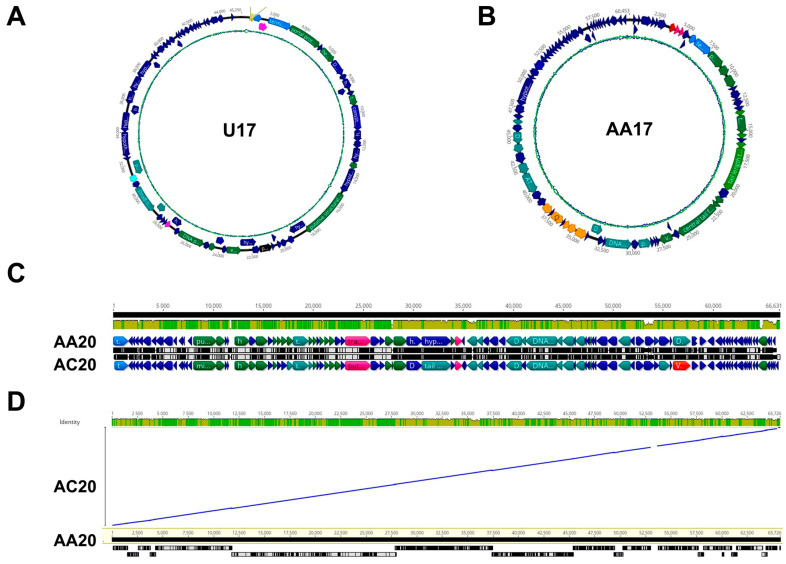
Genomic maps of the four phages. Circularized genomic maps (for ease of viewing, genomes are packaged as linear fragments) of (**A**) U17 and (**B**) AA17. (**C**) Linear genomic maps of AA20 and AC20. Functional assignment of the predicted proteins encoded by each CDS is as follows: hypothetical (navy), morphogenesis (green), transcription and translation (teal), virulence (red), lysis (pink), DNA packaging (blue), regulation and metabolism (black), recombination (dark red), tRNA (yellow), and DNA modifications (orange). (**D**) LASTZ alignment of AC20 genome against AA20 genome. The figure was generated using Geneious Prime.

Phage U17 has a genome size of 45,259 bp and a GC% of 52.5 (Figure 6A). The phage encodes 73 proteins, of which 55 are hypothetical, 14 are assigned to morphogenesis and transcription and translation processes, and two are for lysis. Three tRNAs were identified by tRNAScan-SE and assigned as tRNA-Asn (anticodon GTT), tRNA-Asp (anticodon GTC), and tRNA-Pro (anticodon TGG). The phage genome suggests the phage belongs to LUZ24-like genera, which is one of the seven genera of purely lytic *P. aeruginosa* phages [45]. Interestingly, there is a gene cluster present between the locus tag ABKQ52_48 and 53, which was previously reported as a putative peptidoglycan modification module [46]. The module is predicted to encode putative gamma-glutamyl cyclotransferase (GGCT) protein, ATP grasp enzyme, aminohydrolase, amidoligase, and COOH-NH2 ligase.

Phage AA17 has a genome size of 60,453 bp and a GC% of 56.6 (Figure 6B). The phage encodes 92 putative proteins, of which 62 are hypothetical, 22 are involved in the virion morphogenesis and transcription and translation processes, and two are involved in cell lysis. The closest neighbor is *Pseudomonas* phage Iggy (60.7 kb, 90 CDSs, 56.5% GC, no tRNAs), which is also closely related (94.6%) to *Pseudomonas* phage PBPA162 (MK816297) [47,48]. The phage genome had a gene cluster from ABMZ61_46 to ABMZ61_52 encoding QueD-like 6-pyruvoyl-tetrahydropterin synthase protein, QueC-like queuosine biosynthesis protein, QueE-like radical SAM domain protein, FolE, and DpdA. This gene cluster is reported to be responsible for the 7-cyano-7-deazaguanine (preQ_0_) synthesis and is reported in other *P. aeruginosa*-infecting phages, as well [47,49]. The modified guanine base is suggested to take part in the protection of the phage gDNA from bacterial restriction enzymes.

Phages AA20 and AC20 have a genome of 65,726 bp and 66,242 bp, respectively, and a GC% of 54.8 and 55, respectively (Figure 6C). The AA20 phage encodes 92 putative proteins, of which 60 are hypothetical, 30 are involved in the virion morphogenesis and transcription and translation processes, and two are involved in lysis. Phage AC20 encodes 97 proteins, of which 64 are hypothetical, 29 are involved in the virion morphogenesis and transcription and translation processes, and two are involved in lysis. Due to genomic similarities observed while annotating the genomes, we aligned the AA20 and AC20 genomes by LASTZ, which revealed a 95.6% pairwise identity (Figure 6D).

The phages were studied for therapeutic suitability. The genomic analysis suggests the four phages are strictly lytic due to an absence of identifiable temperate phage repressors. We also used Proksee [50] and Phage Leads [51] to search for antimicrobial resistance or virulence genes in the CARD, VFDB, and ResFinder databases. The analysis revealed an absence of temperate lifestyle, antimicrobial resistance, and virulence-associated genes.

B Comparative Genomics

Based on the NCBI nucleotide data (GenBank) screen using BLASTn, closely related hits were identified for the four phages, with a query coverage for each between 100 and 97%. Phage taxonomy was assigned to genus level as follows:

U17: Viruses; Duplodnaviria; Heunggongvirae; Uroviricota; Caudoviricetes; *Bruynoghevirus*; *Bruynoghevirus* HTN1
AA17: Viruses; Duplodnaviria; Heunggongvirae; Uroviricota; Caudoviricetes; *Iggyvirus*
AA20: Viruses; Duplodnaviria; Heunggongvirae; Uroviricota; Caudoviricetes; *Pbunavirus*
AC20: Viruses; Duplodnaviria; Heunggongvirae; Uroviricota; Caudoviricetes; *Pbunavirus*

**Figure 7:**
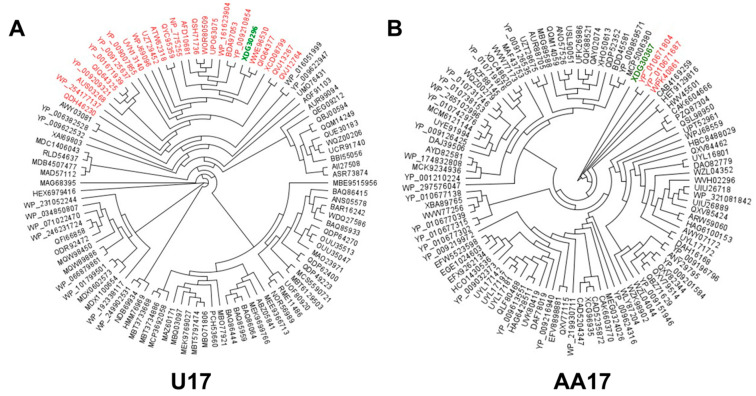
A rooted tree of the large terminase proteins of (**A**) U17 and (**B**) AA17. Each tree was generated using the large terminase protein from each phage against the top 100 BLASTp hits from the NCBI database. A Clustal Omega (v 1.2.3) protein alignment was performed, and the resulting alignment was run with RAxML (v8.2.11) to generate a phylogenetic tree. Green represents large terminase protein of U17 and AA17 and red represents large terminase protein hits with percentage similarity between 95 and 100% to U17 and AA17. The figure was generated using Geneious Prime.

For phages U17 and AA17, further conserved gene-based phylogenetic classification based on the large terminase protein was conducted with the top 100 close relatives based on BLASTp hits from the NCBI database (Figure 7). Based on percentage similarity between 95 and 100%, phage U17 was grouped with 26 other phages, all belonging to the same classification up to the genus level (Figure 7A). On the other hand, phage AA17 was grouped with three phages that only belonged to the same phylogenetic classification up to the genus level (Figure 7B).

Phages AA20 and AC20 had similar taxonomic identification up to the genus level. Upon global alignment with the top seven phage phylogenetic neighbors, they resulted in an Average Nucleotide Identity ANI% of 95.85%, which is above the 95% cut-off calculated by JSpeciesWS [52], which further confirms that they belong to the same species (Table S7).

**Figure 8:**
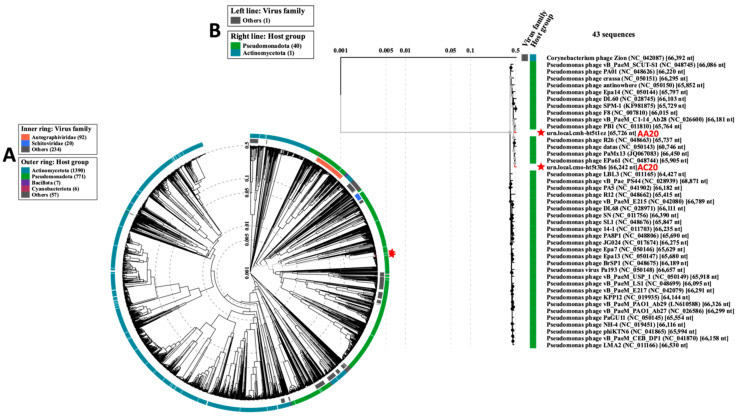
Proteomic tree of phages AA20 and AC20. (**A**) Circular proteomic tree based on genome-wide similarities of phages AA20 and AC20 (marked with red stars) and closely related reference phage genomes. (**B**) Rectangular proteomic tree of phage phages AA20 and AC20 (marked with red stars and labeled in red) and 43 phages with the highest ViPTree SG scores.

From a proteome-based phylogeny perspective, we compared the genome of phages AA20 and AC20 to 2303 sequences of dsDNA prokaryotic viruses using VipTree [53]. Phages with the highest VipTree tBLASTx scores (S_G_) were selected to construct a more specific rectangular proteomic tree. Phages AA20 and AC20 were clustered among other closely related phages whose bacterial hosts belong to the class Pseudomonadota (Figure 8). Phages AA20 and AC20 had an S_G_ score of 0.9218 in relation to each other (Table S8).

**Figure 9:**
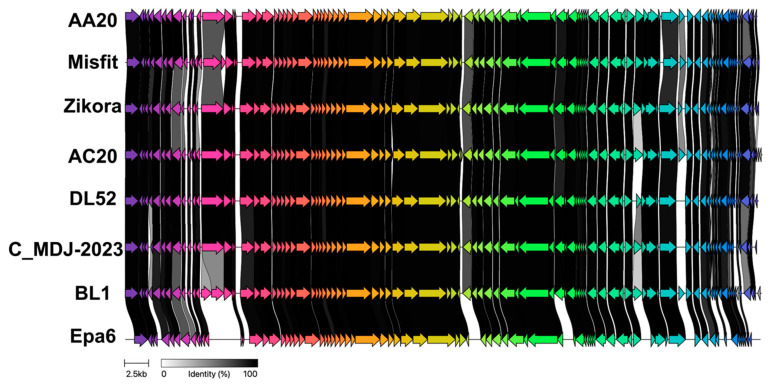
A clinker gene cluster comparison. A comparison of whole genomes for phages AA20 and AC20 against six other closely related phages, with percent amino acid identity represented by greyscale links between genomes. Each similarity group is assigned a unique color.

Next, to check the relationship between phage AA20, phage AC20, and the six closely related phages, a clinker map was generated (Figure 9). This tool is based on protein translations, followed by global alignments between sequences in each cluster. Phages AA20 and AC20 had a low identity percentage in their DNA ligase and two other proteins of hypothetical function. Interestingly, the phages also had a low percentage identity in their tail length tape-measure protein.

C Phages AA17 and AA20 Encode DNA-Modifying Proteins That Confer Resistance to Restriction Enzymes

To test for possible DNA modification, gDNA from each phage was challenged against 16 restriction enzymes (REases) using the maximum incubation time allowable before star activity. The phages showed variable resistance to digestion (Figure S3), with phage AA20 gDNA resisting digestion by ten enzymes, AA17 gDNA was resistant to eight, and phage AC20 was resistant to only seven REases. On the other hand, phage U17 gDNA showed sensitivity to 11 enzymes. The four phages were digested by three enzymes, two of which (MspI and HpaII) target CC sequences and have the four-base-pair (bp) target site CCGG. The third REase, HindIII, targets the following sequence: AAGCTT. Phage AA17 showed an REase-resistant gDNA profile similar to closely related *P. aeruginosa* phage Iggy [47]. Genomic analysis of phage AA17 revealed it encodes proteins with high similarity to the enzymes 7-carboxy-7-deazaguanine synthase and 7-cyano-7-deazaguanine synthase, which are responsible for 7-deazaguanine DNA modifications [49,54].

### 3.3. One-Step Growth Curves for Burst Size Analysis

**Figure 10:**
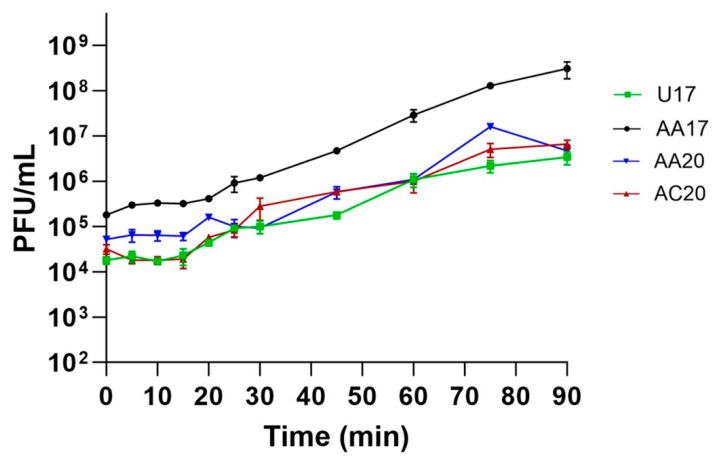
One-step growth curves of the four phages at MOI of 0.001. Data were means of three biological and technical replicates, and error bars represent standard deviation.

One-step growth curve experiments reveal that phages U17 and AC20 have latent periods of 15 min, while phage AA17 has a latent period of 20 min. Phage AA20 has a longer latent period of 30 min. AA17 has a complete cycle of 45 min, while U17 phage’s complete cycle is 90 min. AA20 and AC20 complete their infection cycle within 75 min (Figure 10). For the burst size, AA20 has the largest burst size of the four phages of approximately 311 (±21) PFU per infected host cell, while the burst sizes of U17, AA17, and AC20 are 185 (±24), 27 (±2.7), and 96 (±30), respectively.

### 3.4. In Vitro and In Vivo Virulence Assays

#### 3.4.1. Growth Suppression

**Figure 11:**
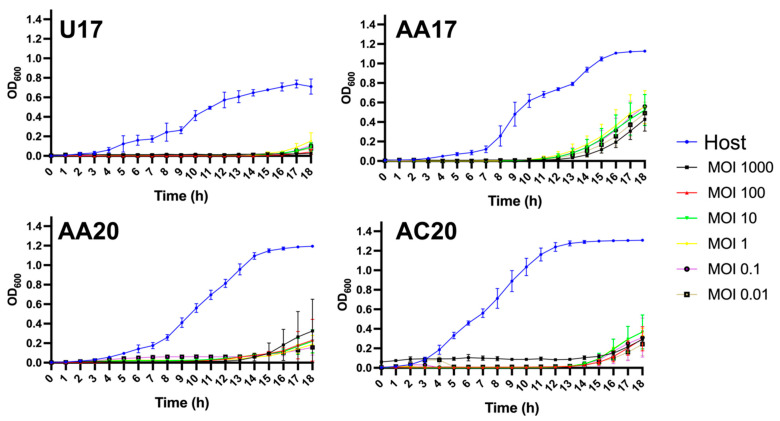
Growth suppression assay. For the four phages, growth suppression kinetics was tested using a combination of the respective bacterial host and six MOIs, starting from 1000 and as low as 0.001. The mean of biological and technical triplicates is represented at each time point, and error bars represent standard deviation.

The host growth suppression capacity of a phage is a critical parameter to study in identifying phage candidates for therapeutic applications. The growth kinetics of the four phages against their respective bacterial hosts was examined at six different MOIs (from 0.001 to 1000) over a period of 18 h. Phage U17 sufficiently suppressed the growth of its host over 18 h, with an MOI as low as 0.01 (Figure 11). Next, both AA20 and AC20 could suppress the growth for 14 h, and finally, AA17, with a suppression of 12 h compared to the untreated bacterial host control (Figure 11).

We also determined the local virulence (VL) and virulence index (Vi) of the four phages. Phages U17, AA17, AA20, and AC20 have high virulence indexes, as follows: 0.98, 0.90, 0.90, and 0.96, respectively (Figure S4). Phage AA17 shows VL variability through the MOIs of 0.01 to 10, with the highest VL with minimal variability at an MOI of 100 (VL = 0.93). The VL of phages U17 and AC20 is the same at MOIs between 0.1 and 100 (VL around 0.98–0.99), then decreases at the highest tested MOI of 1000 (VL 0.95 for U17 and 0.84 for AC20). Finally, phage AA20 shows the highest VL between MOIs of 1 and 1000, with minimal variability (VL between 0.93 and 0.95) (Figure S4).

#### 3.4.2. Phages AC20 and AA20 Delay the Death of *G. mellonella* Larvae Infected with *P. aeruginosa*

**Figure 12:**
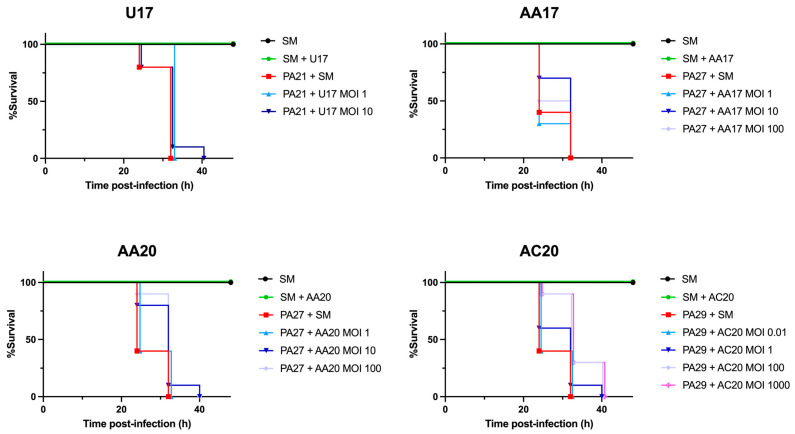
*G. mellonella* phage rescue assays against infection with the corresponding *P. aeruginosa* strain. SM buffer was used as negative treatment control, and larval survival was monitored for a total of 48 h.

Given the effectiveness of each phage at suppressing the growth of the corresponding *P. aeruginosa* strain, a *G. mellonella* larvae model was chosen to assess the effectiveness of each phage in vivo. Groups of 10 larvae in each experiment were first injected with 5 µL of the corresponding diluted bacterial strain, followed by phage administration an hour after the first injection. Bacteria + SM injections showed 100% killing after 32 h for each strain of *P. aeruginosa* (Figure 12). Phages AA20 and AC20 were most effective, showing a dose-dependent delay in larval mortality (Figure 12). At MOIs of 100 and 1000, the administration of phage AC20 showed a significant delay in larval death (*p* < 0.01) and resulted in the survival of three larvae at the 32-h time point when 100% mortality was observed for the bacterial control. Phage AA20 also displayed a significant delay in larval death (*p* < 0.05) at an MOI of 100 but failed to rescue the larvae at the experiment endpoint. Phages AA17 and U17 showed relative improvement when delaying larval death, but both failed to reach statistical significance (Figure 12). Overall, the phages AC20 and AA20 significantly delayed the onset of larval death but failed to ultimately prevent mortality.

### 3.5. Bacterial Twitching Motility Assay

**Figure 13:**
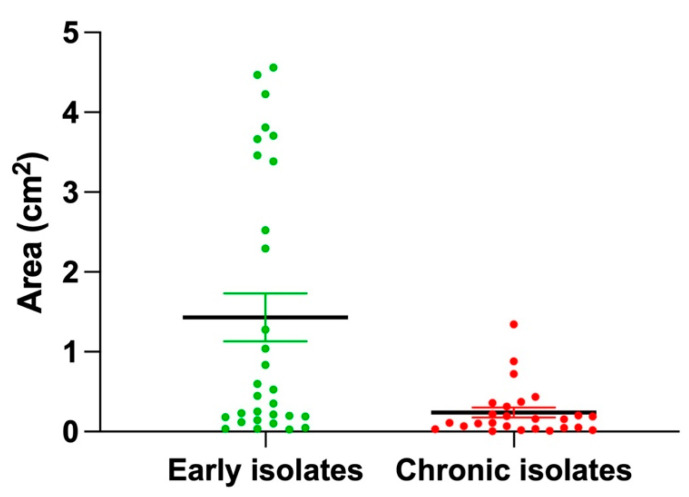
Twitching motility ability of the *P. aeruginosa* panel. The 56 isolates were tested for twitching motility by stabbing 10 uL of bacterial cultures of OD_600_ of 2.0 into the bottom of Eiken agar plates. Each dot shows the area measured at the end of the experiment for each bacterial strain. The black horizontal line represents the mean area in each group. The experiment was performed in biological triplicates, with four plates for each replicate.

Some *P. aeruginosa* strains have the ability to translocate over solid surfaces by a Type IV pilus (T4P)-dependent form of motility known as twitching [55]. Several phages infecting *P. aeruginosa* utilize T4P as a primary cell surface receptor to initiate infection [56,57]. Given the higher sensitivity of early isolates to our phages, we aimed to investigate the potential involvement of T4P as a receptor. To explore this, we assessed the isolate panel for differences in twitching motility. Early isolates exhibited a significantly larger twitching motility area compared to chronic isolates (mean area: 1.432 cm^2^ vs. 0.2397 cm^2^, *p* = 0.006. Statistical analysis was done using *t*-test at a significance level of *p* < 0.05), indicating increased T4P activity in early isolates compared to chronic isolates (Figure 13).

### 3.6. Phages Show a Positive Correlation Trend with Early Isolates in Terms of Infectivity and Twitching Motility Ability

Aiming to test the phage–host interplay, we first examined the correlation between the stage of CF from which isolates were collected and the phage infectivity in solid (spot test) and liquid culture (PHIDA assay). The four phages showed a positive correlation towards early isolates and a negative correlation with chronic isolates in both the spot test and PHIDA assay. The highest significant correlation was for phage AA17 PHIDA score and early isolates (correlation coefficient = 0.266 and Fisher’s Exact test *p*-value 0.004). On the other hand, spot assay results showed no significant association with the isolate type. Since higher phage sensitivity was detected in early isolates, we wanted to examine the possible involvement of T4P as a phage receptor. The twitching motility ability was significantly positively correlated with the early isolate type (correlation coefficient = 0.411 and Fisher’s Exact test *p*-value 0.009). Both the PHIDA and spot assay were able to detect a strong positive correlation between phage AA20 and phage AC20 (correlation coefficient = 0.65 and Fisher’s Exact test *p*-value 0.0004), which was further confirmed by the high similarity in genomic analysis (Figure S5).

## 4. Discussion

The rise in AMR poses a serious global concern and has been declared one of the top ten health threats worldwide. The most recent systematic analysis forecasts a staggering 39.1 million deaths attributable to AMR and 169 million deaths associated with AMR, cumulatively from 2025 to 2050 [58]. One contributor to this crisis is *Pseudomonas aeruginosa*, a high-priority pathogen based on the WHO Bacterial Priority Pathogens List (WHO, 2024). This Gram-negative organism commonly infects immunocompromised individuals, such as CF patients who routinely face significant health challenges throughout their lives [59]. Treatment of *P. aeruginosa* infections in CF individuals often necessitates prolonged antibiotic usage, which often leads to the development of treatment-resistant infections and further antibiotic use. Interest in the use of phages to treat such problematic infections has prompted us to isolate and characterize phages against *P. aeruginosa* for potential therapeutic use.

Six potential phages were isolated from one round of a midi-throughput phage hunt that enables testing of 23 isolates simultaneously. Our protocol screens one sewage sample against 23 bacterial isolates with an overnight incubation in a 96-well plate. The next day, growth-curve data are analyzed, wells with potential phages are collected, and then mini-propagations are performed before further processing the sample for the plaque purification of each phage. The advantage of this hunting protocol is that it suits the basic equipment and the 96/24-well plates available in most microbiology labs. It also minimizes the workload and increases the chances of success in swift phage hunting.

The host range testing identified four phages that collectively covered 62.5% of the tested *P. aeruginosa* panel. Interestingly, phage AA20 was able to solely infect over half of the panel. A previous study on *P. aeruginosa* phages, however, showed a relatively narrow host range to *P. aeruginosa* isolates from CF patients [45]. In this regard, the previous study focused on panels that represent the diversity of the strains, whereas the current study investigated the clinical isolates obtained from different infection (early or chronic) stages. Therefore, it is possible that the high host range observed in our *P. aeruginosa* phages may represent the limited diversity of our panel of clinical *P. aeruginosa* isolates.

As our strain panel consists of early and chronic isolates collected from CF patients, we were interested in studying the general phage sensitivity of each strain panel. To achieve this, we used the phage–host correlation data analytics tool that allows for testing 22 bacterial isolates against four phages in one round. The advantage of testing phages in liquid medium is that it generates quantitative results based on the actual phage–host interaction and kinetics. The agar-based methods, such as spot plating and EOP, are labor-intensive and do not reflect infectivity in liquid culture [25]. Differences in host range were noted for the phages between liquid-based assessments (PHIDA) and spot plating/EOP results. The infectivity pattern was the same; however, an overestimation of the phages’ lytic ability was noticed with spot testing.

The four isolated phages belong to the class *Caudoviricetes* and show the three known podoviral, siphoviral, and myoviral morphotypes. It is reported that most phages infecting *P. aeruginosa* belong to class *Caudoviricetes*, which include all tailed bacterial and archaeal viruses with icosahedral capsids and dsDNA [60].

Genomic analysis of the four phages enabled lifestyle prediction and confirmed their virulent nature. The analysis also showed the absence of antimicrobial resistance genes, making the phages suitable therapeutic candidates. For the U17 phage, further genomic analysis highlighted the presence of a peptidoglycan modification module expressing putative gamma-glutamyl cyclotransferase (GGCT) protein, ATP grasp enzyme, aminohydrolase, amidoligase, and COOH-NH2 ligase. A similar set of genes was also reported in a *Bacillus subtilis* phage [61] and in *P. aeruginosa*-infecting phages such as phiCHU [62]. Since those genes are conserved, with variation in position, domain organization, and number of genes present in other phages, it is highly suggested that they are engaged in a common pathway, specifically to modify the cell wall [46]. The enzymes expressed from those genes are able to synthesize three unusual peptide linkages in the peptidoglycan peptide side chain, which in a phage context helps with the prevention of superinfection. Another suggestion for the presence of this module in lytic phages is that a portion of the bacterial peptidoglycan is replaced with a phage-directed version to create target sites for endolysins to act or even to weaken the cell wall in preparation for lysis [46].

Phage genomes exhibit high diversity, with some viral nucleotides deviating from the canonical bases A, T, C, and G [49]. Those genome modifications play a pivotal role in the incessant arms race between viruses and their hosts. The archaeosine base (G+) found in archaeal tRNAs was recently detected in the *Enterobacteria* phage 9 g genome and proved to impart protection for the phage gDNA against a number of restriction enzymes without blocking the polymerases needed for phage DNA replication [54,63]. Three additional 2′-deoxy-7-deazaguanine modifications, which are all intermediates of the same pathway in viruses, were recently reported, as well: 2′-deoxy-7-amido-7-deazaguanine (dADG), 2′-deoxy-7-cyano-7-deazaguanine (dPreQ_0_), and 2′-deoxy-7-aminomethyl-7-deazaguanine (dPreQ1) [54]. 7-cyano-7-deazaguanine (preQ_0_) is synthesized from GTP by four enzymes (FolE, QueD, QueE, QueC). Additionally, a gene coding for a DpdA homolog is most likely present, although this is not always the case, in 7-deazaguanine-containing phage genomes [54]. The phage DpdA, a paralog of the archaeal guanine-tRNA transglycosylase (arcTGT) enzyme, introduces preQ_0_ in DNA, similar to its bacterial homolog [64]. The biosynthetic pathway commences with GTP, which is transformed to preQ_0_ by the enzymes FolE, QueD, QueE, and QueC. Another pathway is that YhhQ can transport preQ_0_ from the surrounding environment. Finally, the DpdA enzyme exchanges the G bases with preQ_0_ [49]. It has long been reported that homologs of Q synthesis genes are present in phage genomes [54,65,66]. In phage AA17, the gene cluster ABMZ61_46 to ABMZ61_52 encodes the genes for preQ_0,_ which is similar to the dG+ pathway in *Enterobacteria* phage 9 g. Several studies have previously reported phage resistance to restriction enzymes [40,67]. The four phages from this study showed different restriction sensitivity patterns, and this could be attributed to differences in the modification density, which would, in turn, affect the accessibility to restriction sites. Additionally, other modifications could be present but are undetected.

Burst size analysis of the four phages showed variable sizes, with the largest burst size of 311 PFU/cell for phage AA20. Generally, the burst size measurement depends on the host used, the culture medium, and the experimental conditions. Additionally, the procedure used can affect the burst size determination results. For instance, some protocols opt for centrifugation to remove unabsorbed phages, which might lead to lower burst size owing to phage particle adsorption on plastics. AA20 had a comparable burst size to the previously published siphoviral morphotype phage MA-1 that infects *P. aeruginosa*, which has a 330 virions-per-cell burst size [68].

Compared to AA20, phage AC20 showed a lower burst size of 96 PFU/cell. Although phages AA20 and AC20 are highly similar in their genomic characterization, the differences between AA20 and AC20 burst size and latent period could be justified by the non-synonymous single-nucleotide polymorphism detected in their lysis-related genes that leads to seven amino acid differences in the transglycosylase protein and three amino acid differences in the endolysin/lysozyme-like proteins. Additionally, the low percentage identity of the tail tape measure protein could play a role in such discrepancies.

The four tested phages were able to effectively inhibit their host growth for at least 12 h, after which bacteria started to grow again, indicating the emergence of phage resistance. Known resistance mechanisms include the alteration of surface structures to hinder phage attachment, host factor mutations preventing the successful production of virions, and phage defense systems such as CRISPR-Cas [69]. However, there is a fitness cost to phage-induced resistance, such as slower growth rate, decreased virulence, higher susceptibility to host clearance by the immune system, and increased susceptibility to various antimicrobial agents [70,71].

It is generally believed that the larger the burst size, the more potentially useful the phage is for phage therapy. When tested in the *G. mellonella* model, it was somewhat surprising to find that AC20 was able to significantly delay larval mortality to a comparable level as AA20 (Figure 12). Moreover, the protective efficacies in the *G. mellonella* model in our study appear to be poorly correlated to the Vi (AA20 the lowest) or growth suppression assay (U17 the best) data. The reasons for such discrepancies are unknown. It is also possible that the *G. mellonella* model may not be the ideal model for evaluating these phages and/or *P. aeruginosa* strains. The in vivo effectiveness of both AA20 and AC20 phages showed a dose-dependent response, with higher MOIs resulting in an enhanced delay in larval mortality. Nevertheless, these phages were unable to rescue the larvae from the lethal infection. This is consistent with previously reported findings that concluded that the administration of phage prolongs the survival of *G. mellonella* in a dose-dependent manner but not the survival rate [72]. On the contrary, Jeon and Yong reported up to 60% survival rate using their phages in a *G. mellonella* model [73]. We reasoned that the difference might be attributed to the higher burst size and having a different morphotype. For a better outcome, cocktails of the four phages can be tested in vitro, and effective cocktail combinations can then be used in the *G. mellonella* survival assay.

*P. aeruginosa* isolates are known for their twitching motility, which requires the presence of a functional T4P [74]. However, as a CF infection transitions from early to chronic stages, an adaptive behavior is adopted by *P. aeruginosa* isolates to adjust to the pulmonary environment of the CF patient. One noticeable adaptation is the loss of flagella- or pilus-mediated motility [75,76,77]. Interestingly, the early isolates in the tested *P. aeruginosa* panel show a significantly higher twitching motility, which also correlates to their higher scores in the spot test and PHIDA results. Since T4P is a common receptor for diverse *P. aeruginosa* phages [78] and is more highly expressed in early isolates, these isolates may be more susceptible to phages that use T4P as their receptor. Therefore, we attempt to speculate that phage therapy for patients with CF is likely to be more effective if given in the early stage of the disease. In addition, the use of natural or engineered phages targeting other *P. aeruginosa* receptors should be considered in the treatment of late-stage CF patients.

## 5. Conclusions

We isolated four phages targeting *P. aeruginosa* isolates from CF patients. The phages show a host range covering more than half of the tested isolates and have a lytic life cycle with a safe genomic profile and no predicted resistance genes. Additionally, the phages are highly stable at a wide range of temperatures and pH values. It is noteworthy that further engineering or directed evolution of the phages could enhance their in vivo effectiveness. The overall phenotypic and genomic characteristics of the four phages make them promising candidates for therapeutic purposes, either as single phages or in a cocktail.

## Figures and Tables

**Table 1:** Summary of the general genome features of the four phages generated during this study.

Phage	Morphotype	Genome Size (bp)	Number of Predicted CDS	tRNA	G C%	Accession
U17	Podoviral	45,259	73	3	52.5	PP916318
AA17	Siphoviral	60,453	92	0	56.6	PP916319
AA20	Myoviral	65,726	92	0	54.8	PP916316
AC20	Myoviral	66,242	97	0	55.0	PP916317

## Data Availability

Genomic sequences are available with the following accession numbers: *Pseudomonas* phage vB_PaeP_HTN1 (U17); PP916318, *Pseudomonas* phage vB_PaeS_HTN2 (AA17); PP916319, *Pseudomonas* phage vB_PaeM_HTN3 (AA20); PP916316, and *Pseudomonas* phage vB_PaeM_HTN4 (AC20); PP916317.

## Supplementary Materials

The following supporting information can be downloaded at: https://www.mdpi.com/article/10.3390/microorganisms13030511/s1, Figure S1: Host range and phage clustering; Figure S2: Relative efficiency of plating (EOP) of the four phages; Figure S3: RFLP digestion; Figure S4: Local virulence curve of the four tested phages; Figure S5: A colored matrix representing the correlations between the isolate’s type and the spot assay, PHIDA and thetwitching motility assay; Table S1: *Pseudomonas aeruginosa* isolates used in the study and their designation; Table S2: Relative efficacy of plating (EOP) of the four potential phages; Table S3: Annotated CDS of phage U17; Table S4: Annotated CDS of phage AA17; Table S5: Annotated CDS of phage AA20; Table S6: Annotated CDS of phage AC20; Table S7: Average nucleotide identity (ANIb) results of AA20 genome on JSpeciesWS; Table S8: List of phages that have the highest normalized tBLASTx scores (S_G_) to phages AA20 and AC20, calculated by ViPTree.
