# Supplementary material for: Isolation, Characterization, and Genomic Analysis of Bacteriophages Against Pseudomonas aeruginosa Clinical Isolates from Early and Chronic Cystic Fibrosis Patients for Potential Phage Therapy
Source: Microorganisms. 2025 Feb 26;13(3):511. doi: 10.3390/microorganisms13030511 (PMC11945000; doi:10.3390/microorganisms13030511)
Supplement: Supplementary file 1 [file microorganisms-13-00511-s001.zip › PA_SupplementaryFiles_Microorganisms_MDPI.pdf]

Isolation, Characterization, and Genomic Analysis of Bacteriophages Against *Pseudomonas aeruginosa* Clinical Isolates from Early and Chronic Cystic Fibrosis Patients for Potential Phage Therapy

Hanzada T. Nour El-Din [1,*], Maryam Kettal [1], José C. Granados Maciel [1], Greg Beaudoin [1], Umut Oktay [2], Sabahudin Hrapovic [3], Subash Sad [4], Jonathan J. Dennis [2], Danielle L. Peters [1] and Wangxue Chen [1,5]

[1] Department of Immunobiology, Human Health Therapeutics Research Center, National Research Council Canada, Ottawa, ON K1N 5A2, Canada; mkett072@uottawa.ca (M.K.); jose1.granadosm@gmail.com (J.C.G.M.); greg.beaudoin@nrc-cnrc.gc.ca (G.B.); danielle.peters@nrc-cnrc.gc.ca (D.L.P.); wangxue.chen@nrc-cnrc.gc.ca (W.C.)

[2] Faculty of Science, Biological Sciences, University of Alberta, Edmonton, AB T6G 2R3, Canada; uoktay@ualberta.ca (U.O.); jon.dennis@ualberta.ca (J.J.D.)

[3] Aquatic and Crop Resource Development (ACRD) Research Center, National Research Council Canada, Montreal, QC H4P 2R2, Canada; sabahudin.hrapovic@cnrc-nrc.gc.ca

[4] Department of Biochemistry, Microbiology and Immunology, Faculty of Medicine, University of Ottawa, Ottawa, ON K1N 6N5, Canada; subash.sad@uottawa.ca

[5] Department of Biology, Brock University, St. Catharines, ON L2S 3A1, Canada

* Correspondence: hanzada.nourel-din@nrc-cnrc.gc.ca

Supplementary data:

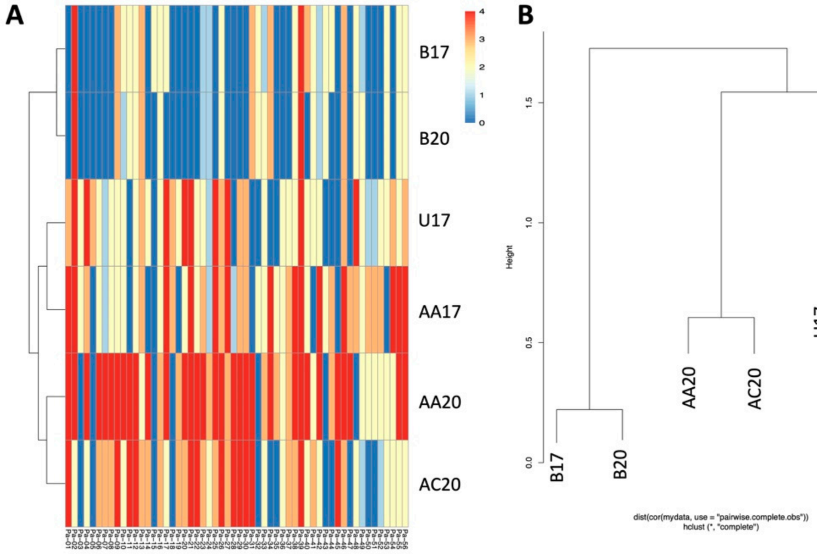

Figure S1: Host range and phage clustering. A. Heat map showing the spot test results of the six tested phages. Figure legend: Red=4, complete clearing, orange=3, clearing with hazy background, yellow=2, substaintial turbidity through the cleared zone, light blue=1, few individual plaques and blue=0, no clearing. **B.** Dendrogram showing the hierarchical clustering based on the distance matrix from the host range correlation between the six tested phages. The figures were generated using R-software.

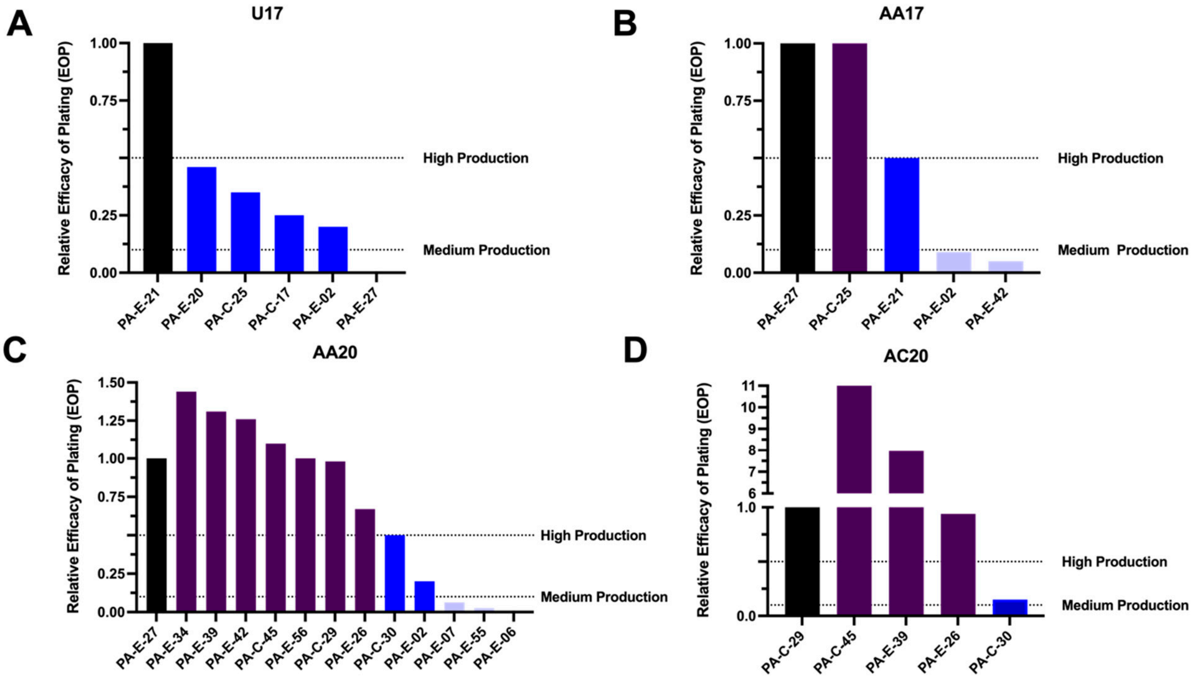

Figure S2: Relative efficiency of plating (EOP) of the four phages. Bar charts representing the EOP of the tested phages against the panel of bacterial strains. The black bar represents the host indicator strain. The relative EOP of the tested panel of bacterial hosts was estimated as a ratio to the host indicator strain. The bars represent the mean of biological duplicate and technical triplicate experiment. The charts were generated using GraphPad Prism (v9) (GraphPad Software Inc., San Diego, CA, USA)

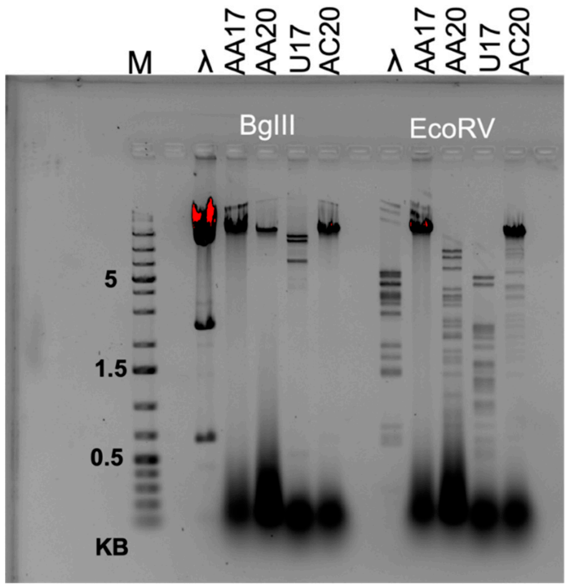

Figure S3: RFLP digestion. 1 μg of phage gDNA was digested using the maximum incubation time at 37 °C and separated on a 1.2 % agarose gel in 1x TAE. Gels showing representative restriction enzymes (BgIII and EcoRV) on the four tested phages and λ control. L: 1 kb Plus GeneRuler DNA Ladder (Thermo Scientific). Images were taken using Bio-Rad Gel Imaging System

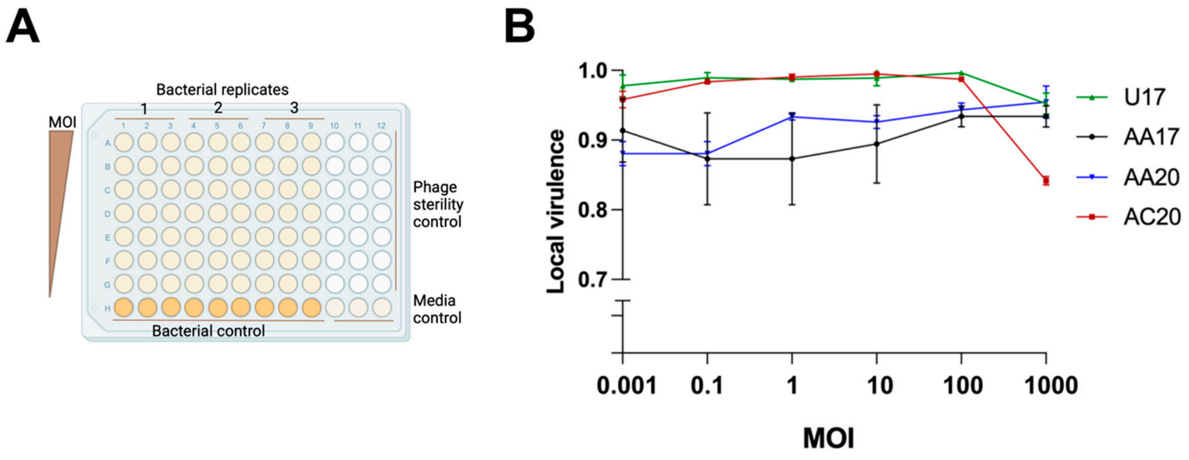

Figure S4: Local virulence curve of the four tested phages. A. 96-well plate layout of the growth inhibition assay. **B.** The OD_{600} of growth reduction curves was measured across six MOIs and virulence curves were generated. The x-axis represents the tested MOIs, and the y-axis represents the local virulence. The chart was generated using GraphPad Prism (v9) (GraphPad Software Inc., San Diego, CA, USA)

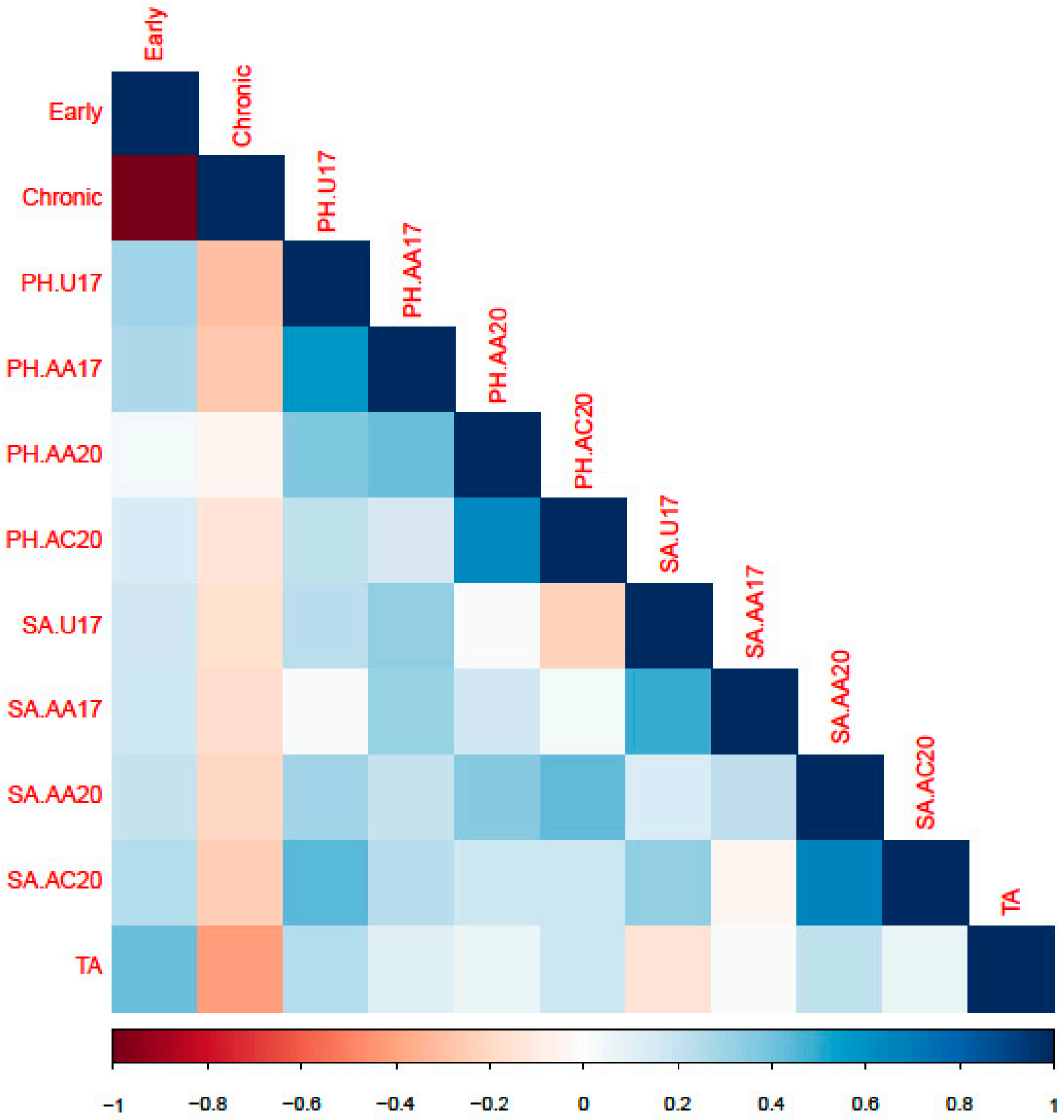

Figure S5: A colored matrix representing the correlations between the isolate's type and the spot assay, PHIDA and twitching motility assay. The color represents Pearson's correlation coefficient, and its intensity represents the coefficient's value (shades of blue are positive correlations and shades of orange-brown are negative correlations). Score: strong twitching motility=3, moderate twitching motility =2, weak twitching motility=1. The figure was

generated using R-software using the corrplot package. Abbreviations: PH, PHIDA; SA, spot assay and TA, twitching assay.

Table S1: ***Pseudomonas aeruginosa*** **isolates used in the study and their designation**

Original code	Internal code	Stage	Original code	Internal code	Stage
**AMT 0116-2**	Pa-01	Early	**AMT-0102-39**	Pa-31	Chronic
**AMT 0116-3**	Pa-02	Early	**AMT-0474-1**	Pa-32	Early
**AMT 0116-27**	Pa-03	Chronic	**AMT-0481-5**	Pa-33	Early
**AMT 0116-29**	Pa-04	Chronic	**AMT-0530-4**	Pa-34	Early
**AMT 0116-31**	Pa-05	Chronic	**AMT-0474-1**	Pa-32	Early
**AMT 0086-2**	Pa-06	Early	**AMT-0481-5**	Pa-33	Early
**AMT 0086-3**	Pa-07	Early	**AMT-0530-4**	Pa-34	Early
**AMT 0086-24**	Pa-08	Chronic	**AMT-0538-2**	Pa-35	Early
**AMT 0102-2**	Pa-09	Early	**AMT-0540-1**	Pa-36	Early
**AMT 0102-38**	Pa-10	Chronic	**AMT-0544-1**	Pa-37	Early
**AMT 0102-40**	Pa-11	Chronic	**AMT-0545-1**	Pa-38	Early
**AMT 0159-1**	Pa-12	Early	**AMT-0547-1**	Pa-39	Early
**AMT 0159-2**	Pa-13	Early	**AMT-0548-1**	Pa-40	Early
**AMT 0159-3**	Pa-14	Early	**AMT-0549-1**	Pa-41	Early
**AMT 0159-25**	Pa-15	Chronic	**AMT-0550-1**	Pa-42	Early
**AMT 0159-26**	Pa-16	Chronic	**AMT-0028-84**	Pa-43	Chronic
**AMT 0159-27**	Pa-17	Chronic	**AMT-0030-74**	Pa-44	Chronic
**AMT 0114-2**	Pa-18	Early	**AMT-0108-26**	Pa-45	Chronic
**AMT 0114-47**	Pa-19	Chronic	**AMT-0118-46**	Pa-46	Chronic
**AMT 0166-1**	Pa-20	Early	**AMT-0130-24**	Pa-47	Chronic
**AMT 0166-2**	Pa-21	Early	**AMT-0209-82**	Pa-48	Chronic
**AMT 0166-3**	Pa-22	Early	**AMT-0302-77**	Pa-49	Chronic
**AMT 0166-4**	Pa-23	Early	**AMT-0321-29**	Pa-50	Chronic
**AMT 0166-23**	Pa-24	Chronic	**AMT-0322-66**	Pa-51	Chronic
**AMT 0166-24**	Pa-25	Chronic	**AMT-0494-9**	Pa-52	Chronic
**AMT 0085-2**	Pa-26	Early	**AMT-0522-5**	Pa-53	Early
**AMT 0085-3**	Pa-27	Early	**AMT-0527-4**	Pa-54	Early
**AMT 0085-22**	Pa-28	Chronic	**AMT-0529-5**	Pa-55	Early
**AMT 0085-23**	Pa-29	Chronic	**AMT-0536-2**	Pa-56	Early
**AMT 0085-24**	Pa-30	Chronic			

Table S2: Relative efficacy of plating (EOP) of the four potential phages.

	U17	AA17	AA20	AC20
**Host range**	9	14	33	12
**II**	3	9	20	7
**Low**	1	2	3	-
**Medium**	4	-	1	1
**High**	1	3	9	4

The EOP is classified into four levels of efficiency from "high production" to "no production" as follow: high (≥ 0.5 - 1), medium (<0.1 - 0.5), low (0.001 - ≤ 0.1) and no production (≤ 0.001). When a phage was able to infect and lyse the host but cannot produce individual plaques or plaques are faint to count at the appropriate dilution, it is given a designation of II.

Table S3: Annotated CDS of phage U17

Table S4: Annotated CDS of phage AA17

Table S5: Annotated CDS of phage AA20

Table S6: Annotated CDS of phage AC20

Table S7: Average nucleotide identity (ANIb) results of AA20 genome on JSpeciesWS

Genome	ANIb [%]	Aligned [%]	Aligned [bp]
**AC20**	95.85	95.64	62,861
**BL1**	95.43	94.65	62,211
**C_MDJ-2023**	98.47	98.04	64,436
**DL52**	96.81	97.54	64,110
**Epa6**	95.39	95.71	62,908
**EPa61**	95.36	94.83	62,326
**Misfit**	96.47	97.90	64,345
**Zikora**	99.02	97.40	64,017

Table S8: List of phages have the highest normalized tBLASTx scores (S_G) to phages AA20 and AC20, calculated by ViPTree

NCBI Accession	Taxonomy ID	Phage name	Genome length	S_G to AA20	S_G to AC20
PP916317.1	-	AC20	66242	0.9218	1
NC_048744	2307269	Pseudomonas phage EPa61	65905	0.9135	0.9485
JQ067083	1175653	Pseudomonas phage PaMx13	66450	0.9212	0.9426
PP916316.1	-	AA20	65726	1	0.9218
NC_007810	347329	*Pseudomonas* phage F8	66015	0.9337	0.9116
NC_048663	2562636	*Pseudomonas* phage R26	65737	0.9089	0.9079
KF981875	1453336	*Pseudomonas* phage SPM-1	65729	0.9285	0.9068
NC_026600	1548917	*Pseudomonas* phage vB_PaeM_C1-14_Ab28	66181	0.9187	0.9066

NCBI Accession	Taxonomy ID	Phage name	Genome length	SG to AA20	SG to AC20
NC_011810	538398	*Pseudomonas* phage PB1	65764	0.9447	0.9058
NC_050150	2719595	*Pseudomonas* phage antinowhere	65852	0.8955	0.905
NC_028745	1640970	*Pseudomonas* phage DL60	66103	0.909	0.9028
NC_050144	2719186	*Pseudomonas* phage Epa14	65797	0.9188	0.8941
NC_050151	2719600	*Pseudomonas* phage crassa	66295	0.8871	0.8921
NC_048626	2498591	*Pseudomonas* phage PA01	66220	0.8595	0.8895
NC_048745	2508865	*Pseudomonas* phage vB_PaeM_SCUT-S1	66086	0.8636	0.8687
NC_041902	1913570	*Pseudomonas* phage PA5	66182	0.8606	0.8573
NC_028939	1542090	*Pseudomonas* phage vB_Pae_PS44	68871	0.8645	0.8507
NC_050143	2719601	*Pseudomonas* phage datas	60746	0.8715	0.8477
NC_011165	549445	*Pseudomonas* phage LBL3	64427	0.8418	0.846
LN610588	1548916	*Pseudomonas* phage vB_PaeM_PAO1_Ab29	66326	0.8257	0.8341
NC_011703	581037	*Pseudomonas* phage 14-1	66235	0.8324	0.8338
NC_042079	2034346	*Pseudomonas* phage vB_PaeM_E217	66291	0.8254	0.8305
NC_026586	1548907	*Pseudomonas* phage vB_PaeM_PAO1_Ab27	66299	0.824	0.8263
NC_048699	2099648	*Pseudomonas* phage vB_PaeM_LS1	66095	0.8103	0.825
NC_041870	1675014	*Pseudomonas* phage vB_PaeM_CEB_DP1	66158	0.8197	0.8232
NC_050146	2719200	*Pseudomonas* phage Epa7	65629	0.8288	0.8228
NC_048662	2562635	*Pseudomonas* phage R12	65415	0.8307	0.8207
NC_048676	2041215	*Pseudomonas* phage SL1	65847	0.822	0.8187
NC_041865	1597972	*Pseudomonas* phage phiKTN6	65994	0.8204	0.8181
NC_019451	1158680	*Pseudomonas* phage NH-4	66116	0.8128	0.8175
NC_011166	549446	*Pseudomonas* phage LMA2	66530	0.8105	0.817
NC_042080	2034347	*Pseudomonas* phage vB_PaeM_E215	66789	0.8245	0.816
NC_048675	2029635	*Pseudomonas* phage BrSP1	66189	0.8225	0.8138
NC_050145	2203191	*Pseudomonas* phage PaGU11	65554	0.8144	0.8123

NCBI Accession	Taxonomy ID	Phage name	Genome length	S_G to AA20	S_G to AC20
NC_028971	1640974	*Pseudomonas* phage DL68	66111	0.8199	0.8114
NC_050149	2743936	*Pseudomonas* phage vB_PaeM_USP_1	65918	0.8189	0.8101
NC_050148	2590837	*Pseudomonas* virus Pa193	66657	0.8188	0.8098
NC_011756	582382	*Pseudomonas* phage SN	66390	0.8148	0.809
NC_017674	749447	*Pseudomonas* phage JG024	66275	0.81	0.8072
NC_050147	2719185	*Pseudomonas* phage Epa13	65680	0.8191	0.8061
NC_019935	763998	*Pseudomonas* phage KPP12	64144	0.8079	0.8046
NC_048806	2848039	*Pseudomonas* phage PA8P1	65690	0.8127	0.8038
